# Supplementary material for: Growth Charts for Shwachman–Diamond Syndrome at Ages 0 to 18 Years
Source: Cancers (Basel). 2024 Apr 5;16(7):1420. doi: 10.3390/cancers16071420 (PMC11010856; doi:10.3390/cancers16071420)
Supplement: Supplementary file 1 [file cancers-16-01420-s001.zip › cancers-2902040-supplementary.pdf]

Supplementary information

Growth Charts for Shwachman-Diamond Syndrome at ages 2 to 18 years

Anna Pegoraro, Valentino Bezzerri, Gloria Tridello, Cecilia Brignole, Francesca Lucca, Emily Pintani, Cesare Danesino, Simone Cesaro, Francesca Fioredda, and Marco Cipolli

Table S1. Number of patients and assessments.

Variable	Age (years)	M	F	Total
Number of patients				
Age				
	0–2	62	43	105
	3–4	35	20	55
	5–6	28	22	50
	7–8	28	25	53
	9-10	26	20	46
	11-12	24	15	39
	13-14	22	11	33
	15-18	23	14	37
Number of assessments				
Weight				
	0–2	147	102	249
	3–4	58	28	86
	5–6	47	32	79
	7–8	39	33	72
	9-10	38	26	64
	11-12	31	17	48
	13-14	28	14	42
	15-18	44	23	67
Height				
	0–2	132	87	219
	3–4	56	28	84
	5–6	46	31	77
	7–8	39	32	71
	9-10	37	25	62
	11-12	31	17	48

	13-14	28	14	42
	15-18	44	22	66
Body mass index				
	0–2	132	87	219
	3–4	56	28	84
	5–6	46	31	77
	7–8	39	32	71
	9-10	37	25	62
	11-12	31	17	48
	13-14	28	14	42
	15-18	44	22	66

M, males; F, females.

Table S2. Measurements of 3rd, 25th, 50th, 75th and 97th percentiles for height, weight, and BMI in the study population.

	Height									
	Males					Females				
Age	C3	C25	C50	C75	C97	C3	C25	C50	C75	C97
0.00	40.71	45.61	48.14	50.55	54.60	39.53	43.90	46.31	48.71	52.95
0.25	45.21	50.56	53.32	55.94	60.36	43.42	48.25	50.89	53.48	58.04
0.50	49.53	55.28	58.24	61.06	65.80	47.21	52.50	55.35	58.13	62.97
0.75	53.57	59.68	62.83	65.82	70.85	50.86	56.59	59.64	62.61	67.71
1.00	57.27	63.70	67.00	70.14	75.42	54.33	60.48	63.73	66.85	72.18
1.25	60.64	67.33	70.77	74.03	79.51	57.58	64.14	67.56	70.82	76.35
1.50	63.68	70.58	74.13	77.50	83.15	60.58	67.52	71.10	74.49	80.18
1.75	66.39	73.47	77.11	80.56	86.35	63.30	70.60	74.31	77.81	83.64
2.00	68.80	76.03	79.74	83.26	89.15	65.74	73.36	77.19	80.78	86.71
2.25	70.96	78.30	82.07	85.64	91.62	67.93	75.85	79.78	83.45	89.45
2.50	72.91	80.36	84.17	87.78	93.83	69.92	78.11	82.13	85.86	91.92
2.75	74.72	82.24	86.10	89.75	95.85	71.73	80.19	84.29	88.06	94.15
3.00	76.40	84.00	87.90	91.58	97.73	73.41	82.11	86.28	90.08	96.21
3.25	77.98	85.66	89.58	93.30	99.49	74.97	83.90	88.14	91.98	98.11
3.50	79.49	87.24	91.19	94.93	101.17	76.45	85.61	89.90	93.77	99.91
3.75	80.94	88.76	92.75	96.51	102.80	77.87	87.25	91.59	95.48	101.62
4.00	82.36	90.25	94.27	98.07	104.39	79.24	88.83	93.22	97.13	103.27
4.25	83.76	91.72	95.78	99.61	105.98	80.55	90.35	94.78	98.71	104.83
4.50	85.16	93.21	97.31	101.17	107.59	81.80	91.80	96.27	100.21	106.33

4.75	86.57	94.71	98.85	102.75	109.23	83.00	93.20	97.70	101.65	107.75
5.00	87.98	96.22	100.40	104.34	110.87	84.14	94.54	99.07	103.03	109.10
5.25	89.36	97.71	101.94	105.91	112.51	85.24	95.82	100.37	104.34	110.39
5.50	90.71	99.16	103.44	107.46	114.12	86.30	97.05	101.63	105.59	111.61
5.75	92.01	100.58	104.90	108.97	115.70	87.31	98.24	102.84	106.80	112.79
6.00	93.25	101.94	106.32	110.43	117.22	88.32	99.41	104.03	107.99	113.95
6.25	94.45	103.25	107.68	111.83	118.69	89.33	100.58	105.22	109.17	115.11
6.50	95.57	104.50	108.98	113.18	120.10	90.35	101.77	106.42	110.38	116.28
6.75	96.64	105.69	110.22	114.46	121.45	91.39	102.97	107.64	111.60	117.47
7.00	97.66	106.82	111.41	115.70	122.75	92.45	104.19	108.88	112.83	118.68
7.25	98.62	107.91	112.55	116.88	123.99	93.53	105.43	110.13	114.08	119.91
7.50	99.54	108.96	113.65	118.02	125.21	94.62	106.67	111.40	115.34	121.14
7.75	100.46	110.00	114.75	119.17	126.42	95.72	107.93	112.67	116.61	122.39
8.00	101.39	111.08	115.89	120.36	127.67	96.83	109.19	113.95	117.89	123.64
8.25	102.38	112.22	117.09	121.61	129.01	97.95	110.46	115.23	119.17	124.90
8.50	103.42	113.41	118.35	122.93	130.41	99.09	111.73	116.52	120.45	126.16
8.75	104.48	114.64	119.65	124.29	131.86	100.24	113.02	117.82	121.75	127.44
9.00	105.56	115.89	120.97	125.67	133.33	101.41	114.33	119.14	123.07	128.74
9.25	106.61	117.12	122.28	127.04	134.79	102.62	115.66	120.49	124.42	130.07
9.50	107.65	118.33	123.56	128.38	136.22	103.86	117.03	121.87	125.80	131.43
9.75	108.64	119.50	124.80	129.68	137.60	105.14	118.44	123.29	127.22	132.84
10.00	109.57	120.60	125.97	130.92	138.92	106.47	119.91	124.77	128.70	134.31
10.25	110.45	121.65	127.08	132.08	140.16	107.85	121.42	126.30	130.24	135.83
10.50	111.27	122.63	128.13	133.18	141.33	109.28	122.98	127.88	131.82	137.40
10.75	112.04	123.56	129.12	134.22	142.44	110.75	124.58	129.49	133.43	139.01
11.00	112.78	124.45	130.08	135.22	143.51	112.25	126.20	131.14	135.08	140.65
11.25	113.50	125.32	131.00	136.20	144.55	113.76	127.84	132.78	136.73	142.30
11.50	114.22	126.20	131.94	137.18	145.59	115.27	129.45	134.42	138.37	143.92
11.75	114.97	127.10	132.90	138.19	146.66	116.74	131.02	136.00	139.96	145.50
12.00	115.76	128.06	133.92	139.25	147.79	118.16	132.54	137.52	141.47	147.01
12.25	116.62	129.08	135.00	140.39	149.00	119.52	133.98	138.97	142.92	148.44
12.50	117.54	130.18	136.17	141.61	150.29	120.82	135.34	140.34	144.28	149.78
12.75	118.53	131.36	137.42	142.92	151.68	122.06	136.64	141.63	145.56	151.04
13.00	119.60	132.62	138.76	144.32	153.16	123.23	137.85	142.84	146.76	152.22
13.25	120.73	133.95	140.16	145.79	154.72	124.33	138.98	143.96	147.87	153.30
13.50	121.90	135.33	141.63	147.32	156.35	125.35	140.01	144.98	148.87	154.27
13.75	123.11	136.75	143.13	148.88	158.01	126.28	140.95	145.90	149.77	155.14
14.00	124.32	138.17	144.63	150.46	159.67	127.14	141.79	146.73	150.58	155.91
14.25	125.51	139.57	146.11	152.00	161.31	127.93	142.56	147.47	151.30	156.59
14.50	126.63	140.90	147.52	153.47	162.87	128.67	143.27	148.16	151.96	157.21
14.75	127.67	142.14	148.83	154.84	164.32	129.38	143.93	148.79	152.57	157.78
15.00	128.60	143.26	150.03	156.09	165.63	130.05	144.57	149.40	153.15	158.31
15.25	129.42	144.26	151.09	157.20	166.81	130.71	145.18	149.98	153.71	158.83
15.50	130.12	145.13	152.01	158.17	167.83	131.37	145.79	150.56	154.26	159.34
15.75	130.69	145.86	152.79	158.99	168.70	132.03	146.40	151.14	154.82	159.85
16.00	131.15	146.46	153.44	159.67	169.42	132.70	147.01	151.73	155.38	160.38
16.25	131.50	146.96	153.98	160.24	170.02	133.38	147.63	152.32	155.95	160.90

16.50	131.77	147.35	154.42	160.70	170.50	134.06	148.26	152.92	156.52	161.44
16.75	131.96	147.66	154.76	161.06	170.89	134.75	148.89	153.53	157.11	161.98
17.00	132.07	147.89	155.02	161.34	171.19	135.44	149.53	154.14	157.69	162.54
17.25	132.12	148.05	155.21	161.54	171.40	136.15	150.18	154.76	158.29	163.10
17.50	132.12	148.15	155.33	161.68	171.54	136.86	150.83	155.39	158.89	163.66
17.75	132.06	148.19	155.40	161.75	171.62	137.57	151.49	156.02	159.50	164.23
18.00	131.98	148.21	155.43	161.80	171.66	138.29	152.14	156.65	160.11	164.80

	Weight									
	Male					Female				
Age	C3	C25	C50	C75	C97	C3	C25	C50	C75	C97
0.00	1.65	2.54	2.93	3.28	3.82	1.50	2.16	2.55	2.97	3.77
0.25	2.31	3.49	4.02	4.50	5.26	2.13	2.99	3.52	4.07	5.12
0.50	2.96	4.40	5.06	5.67	6.64	2.77	3.83	4.47	5.14	6.41
0.75	3.59	5.24	6.03	6.75	7.94	3.43	4.66	5.40	6.17	7.64
1.00	4.19	6.02	6.91	7.75	9.14	4.08	5.46	6.29	7.16	8.80
1.25	4.76	6.73	7.73	8.67	10.25	4.73	6.24	7.15	8.09	9.88
1.50	5.30	7.39	8.47	9.51	11.28	5.35	6.98	7.95	8.96	10.87
1.75	5.82	7.99	9.16	10.29	12.25	5.94	7.66	8.68	9.75	11.76
2.00	6.31	8.56	9.79	11.01	13.16	6.48	8.28	9.35	10.46	12.55
2.25	6.78	9.09	10.39	11.69	14.02	6.98	8.84	9.95	11.10	13.26
2.50	7.23	9.58	10.94	12.32	14.84	7.43	9.36	10.50	11.68	13.90
2.75	7.66	10.05	11.46	12.91	15.63	7.86	9.83	11.01	12.22	14.50
3.00	8.07	10.49	11.94	13.47	16.39	8.25	10.28	11.48	12.72	15.06
3.25	8.47	10.90	12.41	14.01	17.12	8.61	10.69	11.92	13.20	15.59
3.50	8.85	11.30	12.85	14.52	17.84	8.96	11.09	12.36	13.66	16.11
3.75	9.22	11.69	13.27	15.01	18.55	9.29	11.49	12.78	14.12	16.63
4.00	9.58	12.06	13.68	15.49	19.25	9.62	11.87	13.20	14.57	17.15
4.25	9.93	12.42	14.08	15.95	19.95	9.93	12.24	13.61	15.02	17.66
4.50	10.28	12.78	14.47	16.41	20.66	10.24	12.61	14.01	15.46	18.17
4.75	10.63	13.13	14.86	16.87	21.38	10.54	12.97	14.40	15.89	18.67
5.00	10.97	13.49	15.24	17.32	22.10	10.83	13.32	14.79	16.31	19.16
5.25	11.30	13.83	15.62	17.77	22.82	11.12	13.67	15.17	16.72	19.64
5.50	11.63	14.18	16.00	18.21	23.55	11.40	14.00	15.53	17.12	20.09
5.75	11.95	14.52	16.37	18.65	24.27	11.68	14.33	15.89	17.51	20.54
6.00	12.27	14.86	16.75	19.09	25.00	11.96	14.65	16.24	17.89	20.98
6.25	12.59	15.21	17.13	19.54	25.73	12.24	14.98	16.60	18.28	21.42
6.50	12.91	15.57	17.53	20.00	26.47	12.53	15.32	16.97	18.68	21.88
6.75	13.24	15.94	17.94	20.48	27.23	12.83	15.68	17.36	19.10	22.35
7.00	13.59	16.33	18.38	20.99	28.00	13.15	16.05	17.76	19.54	22.86
7.25	13.95	16.74	18.84	21.52	28.78	13.48	16.45	18.20	20.02	23.42
7.50	14.32	17.18	19.33	22.09	29.59	13.82	16.87	18.67	20.54	24.03
7.75	14.71	17.65	19.86	22.69	30.42	14.18	17.33	19.18	21.10	24.70
8.00	15.13	18.15	20.42	23.33	31.27	14.56	17.80	19.72	21.71	25.43
8.25	15.57	18.68	21.02	24.02	32.14	14.94	18.31	20.30	22.36	26.23
8.50	16.03	19.25	21.66	24.74	33.04	15.33	18.84	20.91	23.06	27.10
8.75	16.50	19.84	22.34	25.50	33.95	15.72	19.38	21.55	23.80	28.03

9.00	16.99	20.46	23.03	26.29	34.87	16.11	19.95	22.22	24.59	29.03
9.25	17.48	21.09	23.75	27.09	35.79	16.50	20.53	22.92	25.41	30.09
9.50	17.98	21.73	24.48	27.91	36.71	16.88	21.12	23.65	26.27	31.22
9.75	18.47	22.37	25.22	28.74	37.62	17.27	21.74	24.40	27.17	32.40
10.00	18.96	23.02	25.96	29.57	38.52	17.65	22.36	25.18	28.11	33.65
10.25	19.45	23.67	26.71	30.41	39.41	18.04	23.01	25.98	29.09	34.96
10.50	19.93	24.33	27.46	31.24	40.30	18.43	23.67	26.81	30.09	36.31
10.75	20.41	24.98	28.21	32.07	41.19	18.83	24.35	27.67	31.13	37.71
11.00	20.88	25.63	28.96	32.91	42.08	19.25	25.05	28.54	32.20	39.14
11.25	21.36	26.29	29.71	33.75	42.99	19.68	25.77	29.44	33.28	40.59
11.50	21.84	26.95	30.48	34.60	43.91	20.14	26.50	30.34	34.38	42.05
11.75	22.32	27.62	31.25	35.46	44.85	20.61	27.25	31.26	35.47	43.50
12.00	22.82	28.31	32.04	36.34	45.81	21.12	28.01	32.18	36.56	44.93
12.25	23.32	29.01	32.84	37.24	46.81	21.65	28.78	33.10	37.64	46.31
12.50	23.84	29.72	33.66	38.15	47.84	22.22	29.57	34.02	38.71	47.65
12.75	24.38	30.46	34.51	39.10	48.91	22.82	30.37	34.94	39.75	48.93
13.00	24.93	31.22	35.38	40.07	50.01	23.47	31.18	35.85	40.77	50.15
13.25	25.50	32.00	36.27	41.07	51.16	24.17	32.01	36.76	41.75	51.28
13.50	26.08	32.80	37.20	42.10	52.33	24.92	32.85	37.65	42.70	52.33
13.75	26.68	33.63	38.14	43.16	53.54	25.72	33.71	38.54	43.62	53.29
14.00	27.28	34.47	39.12	44.25	54.78	26.57	34.59	39.42	44.49	54.15
14.25	27.89	35.33	40.11	45.35	56.04	27.48	35.48	40.29	45.33	54.93
14.50	28.51	36.20	41.11	46.47	57.31	28.45	36.38	41.15	46.14	55.61
14.75	29.12	37.08	42.12	47.60	58.57	29.47	37.31	42.00	46.90	56.20
15.00	29.72	37.96	43.13	48.73	59.84	30.54	38.24	42.84	47.64	56.72
15.25	30.31	38.84	44.15	49.86	61.09	31.66	39.19	43.68	48.35	57.17
15.50	30.88	39.71	45.16	50.98	62.33	32.82	40.16	44.52	49.05	57.58
15.75	31.44	40.57	46.16	52.09	63.54	34.00	41.13	45.35	49.73	57.96
16.00	31.97	41.43	47.15	53.19	64.73	35.21	42.11	46.18	50.40	58.30
16.25	32.48	42.27	48.14	54.28	65.90	36.44	43.10	47.01	51.07	58.64
16.50	32.97	43.11	49.12	55.36	67.05	37.67	44.08	47.84	51.73	58.97
16.75	33.43	43.93	50.09	56.42	68.18	38.90	45.07	48.67	52.39	59.30
17.00	33.87	44.75	51.05	57.48	69.30	40.13	46.05	49.50	53.05	59.65
17.25	34.29	45.56	52.00	58.53	70.40	41.35	47.03	50.33	53.72	60.00
17.50	34.68	46.37	52.95	59.57	71.49	42.56	48.00	51.15	54.39	60.37
17.75	35.06	47.17	53.90	60.61	72.57	43.76	48.97	51.98	55.07	60.77
18.00	35.41	47.96	54.85	61.65	73.65	44.95	49.93	52.81	55.75	61.17

	BMI									
	Males					Females				
Age	C3	C25	C50	C75	C97	C3	C25	C50	C75	C97
0.00	9.75	11.83	12.55	13.31	16.03	8.90	11.08	12.49	14.07	17.35
0.25	10.32	12.45	13.21	14.01	16.83	9.76	11.84	13.20	14.71	17.89
0.50	10.85	13.01	13.81	14.65	17.56	10.57	12.56	13.85	15.30	18.35
0.75	11.30	13.49	14.31	15.19	18.18	11.31	13.20	14.43	15.81	18.73
1.00	11.67	13.87	14.72	15.63	18.67	11.96	13.75	14.91	16.22	19.00
1.25	11.97	14.16	15.03	15.97	19.04	12.51	14.19	15.30	16.53	19.16

1.50	12.20	14.38	15.26	16.22	19.32	12.94	14.54	15.58	16.74	19.22
1.75	12.37	14.52	15.42	16.40	19.51	13.27	14.77	15.75	16.85	19.19
2.00	12.49	14.62	15.53	16.52	19.64	13.48	14.90	15.82	16.85	19.06
2.25	12.57	14.68	15.59	16.59	19.71	13.60	14.94	15.81	16.79	18.87
2.50	12.63	14.70	15.62	16.63	19.75	13.65	14.92	15.74	16.67	18.64
2.75	12.67	14.70	15.63	16.64	19.76	13.65	14.86	15.64	16.52	18.40
3.00	12.69	14.69	15.62	16.65	19.76	13.61	14.77	15.52	16.36	18.17
3.25	12.70	14.67	15.60	16.63	19.74	13.56	14.67	15.39	16.21	17.96
3.50	12.69	14.63	15.57	16.60	19.71	13.50	14.58	15.28	16.07	17.79
3.75	12.67	14.59	15.52	16.56	19.66	13.45	14.51	15.19	15.97	17.66
4.00	12.65	14.53	15.47	16.51	19.61	13.42	14.45	15.13	15.91	17.59
4.25	12.62	14.47	15.41	16.46	19.55	13.40	14.42	15.10	15.87	17.56
4.50	12.58	14.40	15.34	16.40	19.49	13.38	14.41	15.09	15.86	17.56
4.75	12.53	14.34	15.27	16.34	19.43	13.38	14.41	15.09	15.87	17.60
5.00	12.49	14.27	15.21	16.27	19.37	13.38	14.41	15.10	15.89	17.65
5.25	12.44	14.20	15.14	16.21	19.31	13.37	14.41	15.10	15.90	17.71
5.50	12.40	14.13	15.08	16.15	19.26	13.35	14.40	15.10	15.91	17.75
5.75	12.36	14.08	15.02	16.10	19.21	13.31	14.37	15.08	15.90	17.78
6.00	12.33	14.03	14.97	16.06	19.18	13.27	14.33	15.04	15.88	17.80
6.25	12.30	13.99	14.94	16.04	19.17	13.21	14.28	15.00	15.85	17.81
6.50	12.29	13.96	14.92	16.03	19.18	13.15	14.23	14.96	15.82	17.83
6.75	12.29	13.95	14.91	16.03	19.21	13.10	14.19	14.93	15.80	17.85
7.00	12.29	13.96	14.92	16.05	19.25	13.06	14.16	14.92	15.81	17.90
7.25	12.32	13.98	14.95	16.09	19.32	13.05	14.17	14.94	15.84	18.00
7.50	12.35	14.01	15.00	16.15	19.42	13.07	14.21	14.99	15.92	18.14
7.75	12.41	14.07	15.07	16.24	19.54	13.11	14.28	15.08	16.04	18.33
8.00	12.49	14.16	15.17	16.37	19.72	13.18	14.37	15.20	16.19	18.57
8.25	12.60	14.29	15.31	16.53	19.94	13.26	14.48	15.34	16.36	18.84
8.50	12.74	14.45	15.49	16.74	20.20	13.34	14.61	15.49	16.56	19.15
8.75	12.90	14.63	15.70	16.97	20.51	13.43	14.74	15.65	16.76	19.47
9.00	13.07	14.83	15.92	17.22	20.84	13.51	14.86	15.81	16.96	19.81
9.25	13.25	15.04	16.16	17.49	21.18	13.56	14.96	15.95	17.15	20.15
9.50	13.43	15.25	16.39	17.75	21.52	13.60	15.05	16.08	17.33	20.48
9.75	13.60	15.45	16.62	18.01	21.86	13.64	15.13	16.20	17.50	20.81
10.00	13.76	15.64	16.83	18.25	22.17	13.66	15.21	16.32	17.68	21.14
10.25	13.90	15.80	17.02	18.47	22.46	13.69	15.29	16.44	17.85	21.47
10.50	14.02	15.95	17.18	18.67	22.72	13.72	15.37	16.56	18.02	21.78
10.75	14.12	16.08	17.33	18.84	22.96	13.77	15.47	16.69	18.20	22.09
11.00	14.21	16.19	17.46	19.00	23.17	13.82	15.57	16.83	18.39	22.38
11.25	14.28	16.28	17.58	19.14	23.36	13.89	15.69	16.99	18.58	22.66
11.50	14.34	16.36	17.67	19.26	23.53	13.98	15.83	17.15	18.78	22.92
11.75	14.39	16.42	17.76	19.36	23.68	14.08	15.98	17.33	18.98	23.15
12.00	14.42	16.48	17.83	19.45	23.82	14.20	16.13	17.51	19.18	23.35
12.25	14.45	16.52	17.89	19.54	23.94	14.33	16.30	17.69	19.38	23.53
12.50	14.47	16.56	17.95	19.62	24.06	14.47	16.48	17.89	19.58	23.68
12.75	14.50	16.61	18.01	19.70	24.19	14.62	16.66	18.08	19.78	23.81
13.00	14.52	16.65	18.07	19.78	24.32	14.78	16.85	18.27	19.97	23.92

13.25	14.56	16.71	18.15	19.89	24.47	14.95	17.04	18.47	20.15	24.01
13.50	14.61	16.79	18.25	20.01	24.65	15.13	17.24	18.66	20.33	24.08
13.75	14.69	16.90	18.38	20.17	24.86	15.31	17.43	18.85	20.49	24.13
14.00	14.78	17.02	18.53	20.35	25.12	15.50	17.63	19.04	20.65	24.16
14.25	14.89	17.18	18.71	20.57	25.41	15.70	17.83	19.22	20.79	24.17
14.50	15.02	17.35	18.92	20.81	25.73	15.90	18.02	19.39	20.93	24.17
14.75	15.17	17.54	19.14	21.07	26.07	16.10	18.21	19.55	21.04	24.14
15.00	15.32	17.74	19.37	21.35	26.44	16.31	18.39	19.71	21.15	24.10
15.25	15.48	17.95	19.62	21.64	26.82	16.52	18.57	19.86	21.25	24.06
15.50	15.63	18.16	19.87	21.93	27.20	16.74	18.76	20.00	21.35	24.02
15.75	15.79	18.36	20.11	22.22	27.58	16.96	18.94	20.15	21.44	23.98
16.00	15.94	18.57	20.35	22.50	27.95	17.18	19.12	20.29	21.54	23.94
16.25	16.07	18.76	20.58	22.78	28.31	17.40	19.29	20.43	21.62	23.90
16.50	16.20	18.94	20.80	23.04	28.66	17.60	19.45	20.55	21.69	23.86
16.75	16.32	19.11	21.01	23.29	28.99	17.80	19.60	20.66	21.75	23.81
17.00	16.42	19.27	21.20	23.52	29.30	17.99	19.74	20.75	21.80	23.76
17.25	16.52	19.42	21.39	23.76	29.61	18.17	19.86	20.84	21.85	23.70
17.50	16.62	19.57	21.58	23.99	29.92	18.34	19.98	20.93	21.89	23.65
17.75	16.71	19.72	21.77	24.22	30.23	18.50	20.09	21.00	21.92	23.59
18.00	16.80	19.87	21.95	24.45	30.53	18.66	20.20	21.07	21.95	23.54
